# *In-Silico* Structural and Functional Characterization of a *V. cholerae* O395 Hypothetical Protein Containing a PDZ1 and an Uncommon Protease Domain

**DOI:** 10.1371/journal.pone.0056725

**Published:** 2013-02-18

**Authors:** Avirup Dutta, Atul Katarkar, Keya Chaudhuri

**Affiliations:** 1 CSIR-SRF, Molecular and Human Genetics Division, CSIR - Indian Institute of Chemical Biology, Kolkata, West Bengal, India; 2 ICMR-SRF, Molecular and Human Genetics Division, CSIR - Indian Institute of Chemical Biology, Kolkata, West Bengal, India; 3 Chief Scientist, Molecular and Human Genetics Division, and Head Academic Affairs Division, CSIR - Indian Institute of Chemical Biology, Kolkata, West Bengal, India; Russian Academy of Sciences, Institute for Biological Instrumentation, Russian Federation

## Abstract

*Vibrio cholerae*, the causative agent of epidemic cholera, has been a constant source of concern for decades. It has constantly evolved itself in order to survive the changing environment. Acquisition of new genetic elements through genomic islands has played a major role in its evolutionary process. In this present study a hypothetical protein was identified which was present in one of the predicted genomic island regions of the large chromosome of *V. cholerae* O395 showing a strong homology with a conserved phage encoded protein. *In-silico* physicochemical analysis revealed that the hypothetical protein was a periplasmic protein. Homology modeling study indicated that the hypothetical protein was an unconventional and atypical serine protease belonging to HtrA protein family. The predicted 3D-model of the hypothetical protein revealed a catalytic centre serine utilizing a single catalytic residue for proteolysis. The predicted catalytic triad may help to deduce the active site for the recruitment of the substrate for proteolysis. The active site arrangements of this predicted serine protease homologue with atypical catalytic triad is expected to allow these proteases to work in different environments of the host.

## Introduction

*Vibrio cholerae*, the most notable member of the *Vibrionaceae* family is the etiological agent of epidemic cholera, causing a severe and sometimes lethal diarrheal disease. *V. cholerae* is classified into two serogroups: O1 and nonO1. So far, the toxigenic strains of serogroups O1 and O139 have been found to cause cholera epidemics. There are two biotypes of *V. cholerae* O1, Classical and El Tor. There have been seven major pandemics since 1817. Isolates of the sixth pandemic were of O1 classical biotype [1].

The complete genome of *V. cholerae* classical biotype has been sequenced, which revealed that the genome is composed of two chromosomes, the large and the small chromosome [2]. Cumulatively 3875 genes have been identified. However, 1402 open reading frames, code for hypothetical proteins, the functions of which are not known.

*V. cholerae* infection is noninvasive. In this organism, the two major virulence factors cholera toxin (CT) and toxin corregulated pili (TCP) have been reported to be encoded on mobile genetic elements. Gene acquisition and other genomic alterations, by the mechanism of Horizontal gene transfer have always played a critical role in the adaptive evolution of prokaryotes. Genomic Islands (GIs) in prokaryotic genomes often contain horizontally transferred genetic materials as evident from the presence of integrase, transposons, phage mediated genes, etc. in these islands [3]–[5]. These genomic islands are therefore of critical importance in the evolution of the prokaryotic genomes, their pathogenicity and other special function.

The *ctxAB* genes coding for CT are encoded on a filamentous bacteriophage CTXφ [6]. TCP, an essential colonization factor, was originally designated as part of a pathogenicity island named Vibrio pathogenicity island VPI, but this island has later on been proposed to be the genome of a filamentous phage, VPIφ [7]. Clinical trials on volunteers using vaccine strains of *V. cholerae* in which several toxin genes including the cholera toxin were eliminated were performed. Results of those trails showed mild to moderate diarrhea in the subjects clearly suggesting that there are yet to be determined virulence factors in the *V. cholerae* genome [8].

In order to survive distinct stress situations and prevent the accumulation of misfolded and aggregated proteins, all cells employ an efficient protein quality control system consisting of molecular chaperones [9], [10] in order to prevent cellular malfunctions and even cell death [11], [12]. The high temperature requirement A (HtrA) family of proteases are involved in the key aspects of protein quality control [13]. In *Escherichia coli* they have been reported to monitor the proper folding and the functioning of the proteins in cell envelope and the periplasm [14]. HtrA proteases consists of a chymotrypsin-like serine protease as their catalytic domain with one or two C-terminal PDZ domains [15], [16]. The PDZ domains are responsible for substrate binding and controlling protease function. In case of *E. coli*, three HtrA proteases, DegS, DegP and DegQ are responsible for the protein quality control [17]. Prokaryotic HtrAs have been reported to be involved in not only protein quality control but in pathogenicity as well [18]–[25]. A similar kind of HtrA - protease DO is present in *Vibrio cholerae* O395 which is a homologue of the DegQ protein of *Escherichia coli* H299. Studies have shown that *htrA* mutant in many Gram negative pathogens are attenuated in animal models and can act as live vaccines [14]. A vaccination study indicated that the purified recombinant DegQ protein acted as a protective immunogen conferring protection upon fish against infection by *V. harveyi* [26].

In the present study a hypothetical protein had been identified which was present in one of the predicted genomic island regions of the large chromosome of *V. cholerae* O395. This hypothetical protein showed strong homology with a conserved phage encoded protein. Homology modeling study indicated that the hypothetical protein was an unconventional and atypical serine protease belonging to HtrA protein family. The predicted 3D-model of the hypothetical protein revealed that it had a serine residue at its catalytic center which utilizes a single catalytic residue for proteolysis. The predicted catalytic triad may constitute the active site for the recruitment of the substrate for proteolysis. Recently revealed crystallographic structure of DegQ and DegP with higher order oligomers suggested that signaling cascade leading to protease activation of 12- and 24-mer HtrA complex was highly conserved and depended on precise positioning of PDZ1 domain upon substrate engagement. The active site arrangements of this predicted serine protease homologue with atypical catalytic triad is expected to allow these proteases to work in different environments of the host.

## Results

### Identification of genomic islands in *V. cholerae* O395

Co-ordinates of statistically significant horizontally acquired genomic segments of *V. cholerae* O395 were determined by *Design-Island* [27]. A customized Perl script was used to mark out the coding regions from the predicted Genomic Islands (GIs) using the protein table as the reference available at the NCBI database. The results showed that after the *refinement phase* the GIs covered ∼44% of the large chromosome and ∼41% of the small chromosome (Data not shown). *Design-Island* identified all the known GIs of *V. cholerae* Classical O395, such as CTXφ, VPI-1, VPI-2 [28]–[30]. Along with the known ones, a number of genomic segments, which has the potential of being GIs, were also identified. Some of these new segments were flanked by transposase or integrase genes or had phage or potential phage related genes. The Perl script developed for the visualization of the putative GIs used the coordinates obtained from the output of *Design-Island* to generate a circular map of each chromosome (Figure S1), the newly identified regions are shown in supplementary figures (Figure S2A & Figure S2B).

Our study revealed a distinct GI region in the large chromosome of *V. cholerae* Classical strain O395, which was absent in the El Tor strain N16961 of *V. cholerae*. This unique cluster consisted of a number hypothetical proteins, phage related proteins and other biosynthetic and transferase like proteins. Conserved domain analysis of these hypothetical proteins showed that many of these had domains of phage related proteins, clearly indicating the possibility of gene acquisition from phages. Among these hypothetical proteins one having locus tag VCO395_1035, came up which did not show any hit with any of the conserved domains of known protein functions as determined by CDD search analysis. However this protein emerged as a potential periplasmic protein when checked for possible localization using the HSLpred [31], CELLO [32], [33] and the SubLoc v1.0 servers [34] (vide Subcellular Localization section).

### Structure Functional Analysis of the Protein VCO395_1035

To determine the possible function of *V. cholerae* VCO395_1035, the sequence was subjected to comparative protein structure modeling using the target protein sequence as query for different servers described in materials and methods. Significant hits were obtained for the ModWeb server [35] which retrieved the crystal structure of the protease along with the PDZ1 domain of DegQ from *Escherichia coli* (PDB ID: 3STJ). The alignment coverage region for target residue (17–207) showed the 34% sequence identity with template 3STJ residue 152–309.

### Comparative Sequence Analysis and Alignment

**Figure 1:**
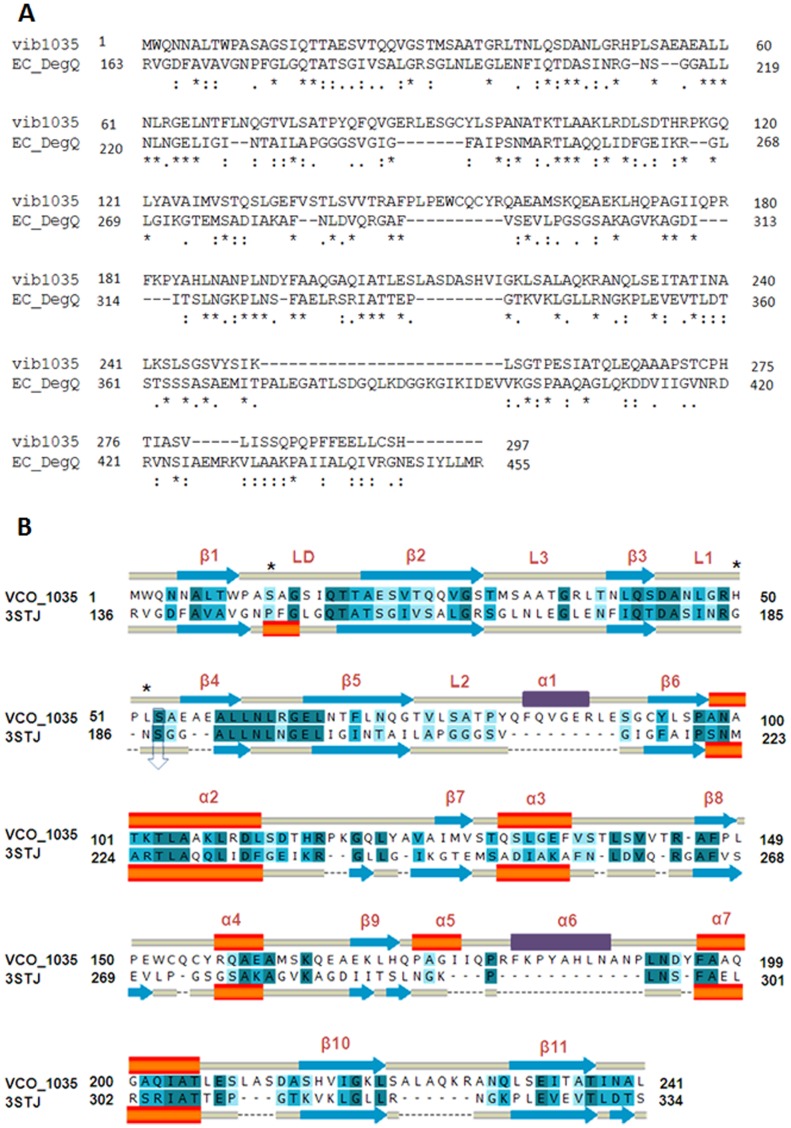
Sequence alignment of VCO395_1035 with *E. coli* DegQ. A. **Sequence alignment of the query (vib1035) and the** ***E. coli*** **DegQ (EC_DegQ).** The ‘*’ indicate the conserved amino acids; ‘:’ represents similar group of amino acids. B. **Sequence alignment used for 3D-modeling of VCO395_1035 using** ***E. coli*** **DegQ as template (PDB ID: 3STJ).** The blue arrows indicate β sheets, orange bars indicate helix and the yellow bars indicate loops. The deep blue color indicates identical amino acids; lighter blue colors indicate similar and weakly similar amino acids. The two major loop modeled to their corresponding secondary structure were shown in violet color. The predicted catalytic triad residue Ser12-His50-Leu52 indicated by ‘*’ and conserved Ser53 residue with DegQ Ser187 which is one of catalytic triad residue of DegQ of *E. coli* indicated by down arrow.

The hypothetical protein VCO395_1035 when aligned with *E. coli* DegQ, shared 25.7% identity and 40.7% similarity as shown in Figure 1A. DegQ contains a protease domain and two distinct domains, PDZ1 and PDZ2 at 258–349 and 355–445 amino acid residues respectively. The target sequence showed maximum conserved residue in the coverage of the PDZ1 domain of the protease chain. For the PDZ2 domain, the residues were showing low identity and similarity. PDB structure of 3STJ lacked the PDZ2 domain coordinate hence for the further modeling and analysis was restricted to Protease+PDZ1 domain. The first 241 residues were selected, in which conserved residues were aligned properly with the functionally essential regions of the protein template. The proposed alignment for homology modeling of VCO395_1035 is shown in Figure 1B.

### Homology Modeling of VCO395_1035 and Validation

The three-dimensional structure of a hypothetical VCO395_1035 from DegQ of *Escherichia coli* (PDB ID: 3STJ chain A, at 2.6 Å resolution) was used as template for homology modeling. The Comparative modeling of VCO395_1035 was performed using a restrained-based approach implemented in MODELLER9v6 [36]. A set of 10 models for each target protein was constructed. The resulting three-dimensional models of VCO395_1035 were sorted according to scores calculated from discrete optimized protein energy (DOPE) scoring function [37]. The final model that shared the lowest Root Mean Square Deviation (RMSD), relative to the trace (Cα atoms) of the crystal structure was selected. The final deviations in the protein structure geometry was regularized by energy minimization with the GROMOS96 force field [38] using Deep View [39] by applying 200 steps steepest descent algorithm and 200 steps conjugate gradients algorithm. The final model had 2 major loops, which arose due to insertion (Figure 1B). The two major loops, one from protease domain (residue 79–89; TPYQFQVGERL) and another from PDZ1 domain (residue 176–189; IIQPRFKPYAHLNANPL) were submitted on FALC-Loop webserver for predicting the local structure of loops [40]. The server was used to construct loop region and to refine unreliable loop region in homology modeling by employing an *Ab-initio* loop modeling method FALC (fragment assembly and analytical loop closure) of designed sequence [41]. The output modeled loop after gradient minimization of FALC which had low DFIRE energy, L-RMSD (Cα RMSD of loop after superimposition of loop structures), A-RMSD (Cα RMSD of loop at the fixed framework) and C-RMSD (Cα RMSD of loop of protein structure) was selected and complete loops assembled model further allowed for energy minimization with 100 steps steepest descent and 100 steps conjugate gradients. The final model was validated by using PROCHECK [42] and TM-align [43].

### Validation of Homology Model of VCO395_1035

The quality of backbone conformation of model was assessed by PROCHECK for reliability [42]. The observed Psi-Phi pairs had, 82.7% of residues in most favored regions, 15.7% residues in additional allowed regions, 1.1% residues in generously allowed regions and 0.5% residues in disallowed regions as shown in Figure S3 and values shown in Table S1 indicated a good quality model.

The members of HtrA family (DegP, DegQ and DegS) protease exhibit highly extensive ordered secondary structure of α-helix and β-sheet. The final refined model of VCO395_1035 was superimposed with template by using TM-align server [43]. A calculated root-mean-square deviation (RMSD) value of 1.16 Å and TM-score of 0.797 was normalized by length of the template protein. The superimposition of model to the template was shown in Figure S4.

### Characterization of Homology Model of VCO395_1035

**Figure 2:**
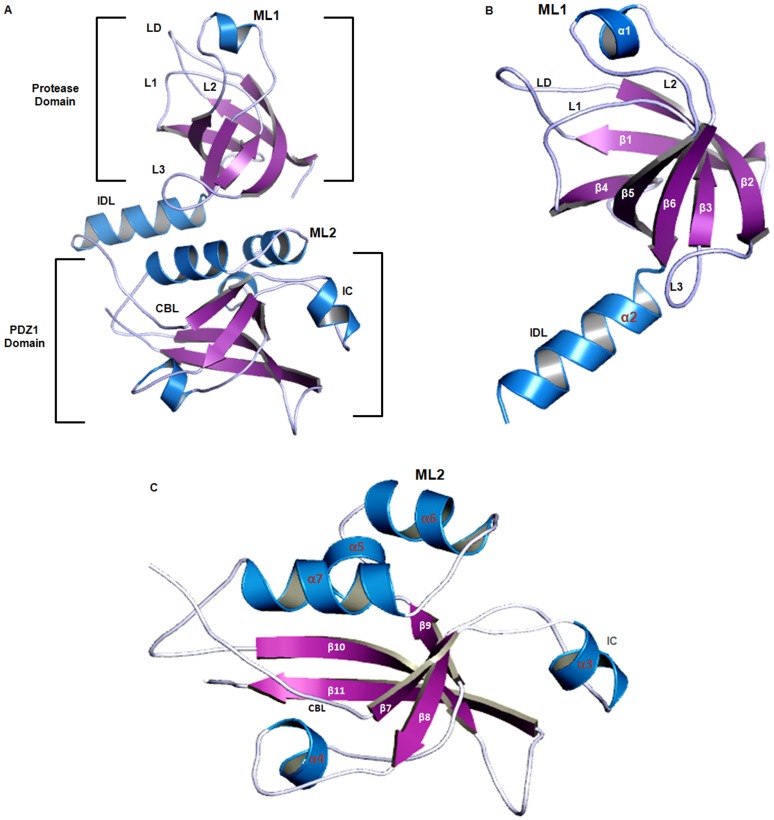
Characterization of Homology Model of VCO395_1035. A. The cartoon representation of 3D modeled structure of VCO395_1035 using PDB ID: 3STJ. Helix (blue), sheets (Purple) and loops (Sky Blue). B. The β-barrel like structure of protease Domain of VCO395_1035 showing active site loops LD: Activation loop, L1: Oxyanion loop, L2: Substrate specificity and L3: Regulatory loop along with interdomain linker (IDL) helix. ML 1: Modeled loop 1 in Protease domain (residue 79–89) on FALC-Loop server indicated as α1-helix. C. The PDZ1 Domain of VCO395_1035, showing flexible carboxylate binding loop (CBL) and interacting clamp (IC). ML 2: Modeled loop 2 in PDZ1 domain (residue 176–189) on FALC-Loop server indicated as α6-helix.

The 3D model of VCO395_1035 using the template 3STJ, consisted of two domains, namely a protease domain and PDZ1 domain (Figure 2A). The 3D model of VCO395_1035 using the template 3STJ, consisted of two domains, namely a protease domain and PDZ1 domain (Figure 2A). In order to characterize the model, structural motif and mechanistically important loops were assigned to build the final 3D model of VCO395_1035. The final model consisted of 11β-beta-sheets and 7α-Helix, the details of which are presented in Table S2.

Protease domain (residue 1–111) consisted of 6β-sheets arranged anti-parallel to form β-barrel like structure and their positions were stabilized by the corresponding loops which may take part in activation mechanism and active site formation (Figure 2B). PDZ1 domain of 3D-model VCO395_1035 (residue 112–241) consisted of 5β-sheets and 5α helix adopted a β-sandwich fold (Figure 2C). The flexible loop of PDZ1 domain of VCO395_1035 contained the highly conserved “carboxylate binding loop” (CBL) (residue 119–122).

### Active Site

**Figure 3:**
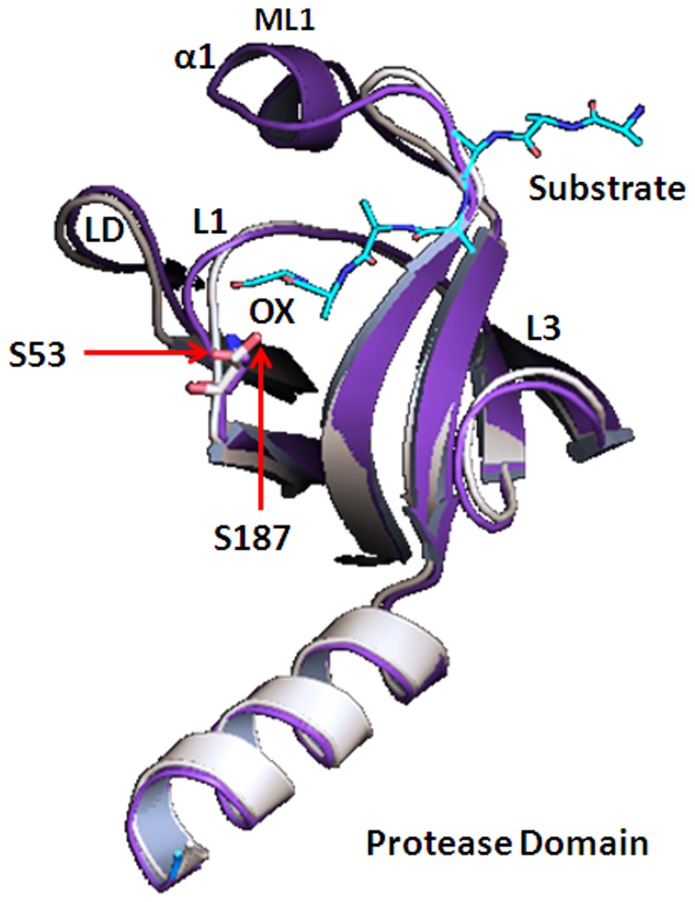
Structural alignment of protease domain. The cartoon representation of protease domain of model VCO395_1035 (magenta) aligned with template 3STJ (light orange) showing conserved Ser53 with DegQ Ser214 which is one of catalytic triad residue of DegQ along with substrate (cyan) bound to active site in Oxyanion hole(ox).

**Figure 4:**
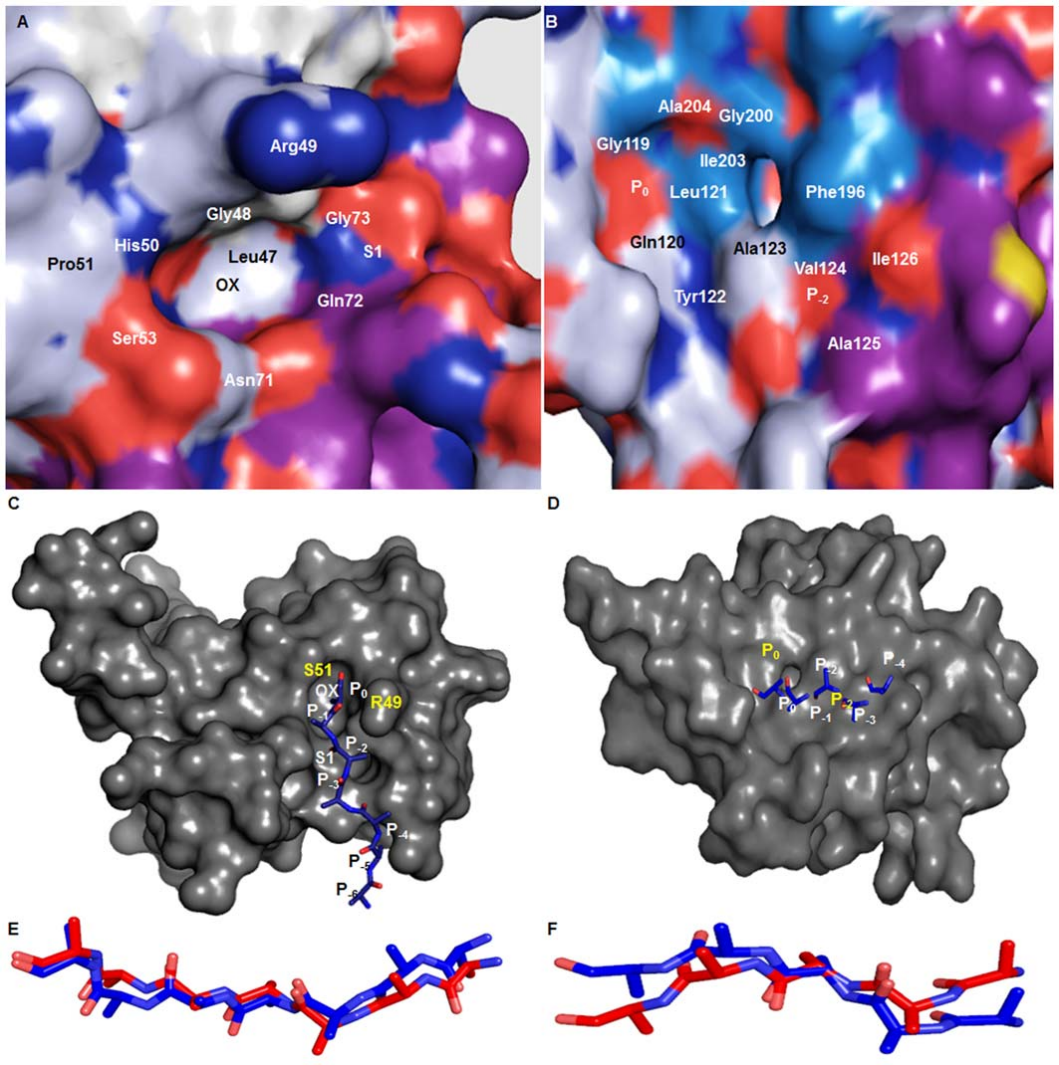
Active site and Protein-substrate interaction using Hex 5.0. A. The surface view of protease domain containing active site showing the oxyanion hole and properly oriented shallow S1 hydrophobic pocket. B. The surface view of PDZ1 containing hydrophobic binding groove formed by CBL and α7-Helix showing shallow P_0_ and P_−2_ substrate binding pocket. C. The C-terminal of poly-alanine peptide substrate (blue) docked into active side of protease domain. D. The C-terminal of poly-alanine peptide substrate (blue) docked into active side of PDZ1 domain via β-aggumentation. E. The superimposition of substrate docked into the protease active site (blue) with respective to template (3STJ) substrate (red). F. The superimposition of substrate docked into the active site PDZ1 domain (blue) with respective to template (3STJ) substrate (red).

The protease domain of VCO395_1035 3D-model showed well-defined active site. The alignment of VCO395_1035 with active state DegQ clearly showed conserved active site containing Ser53 (Figure 3). The active site is formed by the proper adjustment of Ser53, Oxyanion hole and the S1 specificity pocket. The amide linkage between Gly48 and Arg49 of loop L1 enabled the Arg49 carbonyl oxygen to interact with the amide nitrogen of Ala13 of loop LD thus allowing the formation of Oxyanion hole. The orientation of the residues Leu47, Gln72, Gly73 and Thr79 form the shallow hydrophobic S1-specificity pocket. The residues which were actively participating in formation of active site containing Oxyanion hole, S1 pocket and void were shown in Figure 4A, Table S3. The PDZ1 domain of VCO395_1035 containing the deep binding clef, was formed by the Carboxylate binding loop (CBL), β7-sheet and α7-helix. The two hydrophobic pockets were formed P_0_ and P_−2_. The residues involved in the formation of hydrophobic binding pockets were shown in Figure 4B, Table S3.

### Docking Study

The protease domain and PDZ1 domain were predicted to be involved in substrate binding through the recognition of C-terminal residue of the substrate molecule. In order to check the mode of binding of the substrate molecule in the predicted 3D-model of VCO395_1035, two polyalanine oligopeptides from template (PDB ID: 3STJ) were selected. The protease domain was docked by seven residue polyalanine peptide molecule substrate and PDZ1 domain was docked by five residue polyalanine peptide molecule substrate. The docking was performed by Hex 5.0 software [44] using the reference of the template substrate molecule complex. The best dockpose was then refined and analyzed. The docking study showed active site of the protease domain interacted with substrate molecule by β-augmentation. The residues involved the specific binding of incoming substrate molecule with Ser53 as shown in Table S4. The C-terminal P_0_ residue of substrate interacted with Ser53 and P_−2_ residue with the S1 specificity pocket (Figure 4C). The second peptide was bound to PDZ1 domain, the groove of PDZ1 domain was formed between α7-helix and adjacent to β7-strand, allowing the C-terminal ends of the substrate molecule to serve as an extra β-strand added to the β-sheets. The C-terminal P_0_ residue of the polyalanine was bound to the P_0_ pocket and P_−2_ pocket of PDZ1 active site by residue shown Table S4 and Figure 4D. After docking with the substrate molecule, RMSD deviation was calculated which showed that 3D-model had deviated from 1.16 Å to 1.18 Å, suggesting that the mode of binding of substrate molecule with respective binding site were feasible and correct. The docking pose of substrate molecule with respective to the template substrate were shown in Figure 4E & F.

### Catalytic Triad in the Protease Domain

**Figure 5:**
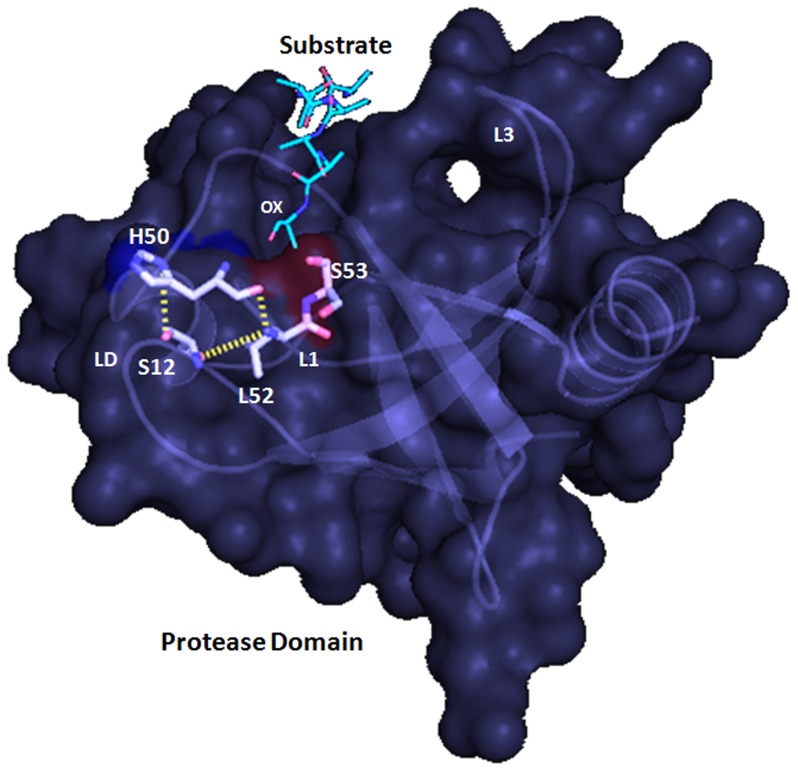
Predicted catalytic triad of model VCO395_1035. The cartoon and surface representation of model VCO395_1035 showing predicted catalytic triad residue Ser12-His50-Leu52 along with along with substrate (cyan) bound to active site in Oxyanion hole (ox).

**Figure 6:**
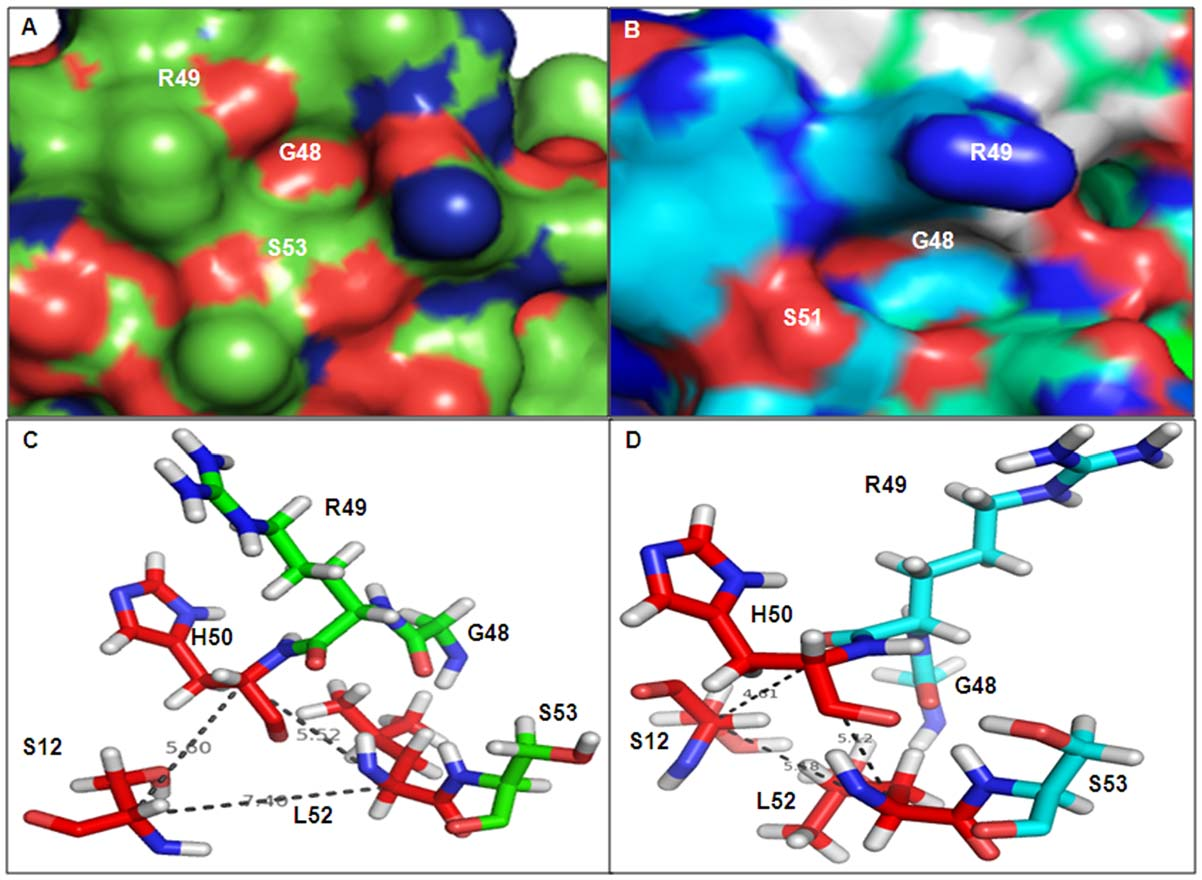
Organization of Active and Inactive form of serine containing proteolytic active site and Catalytic triad. A. Substrate binding site of inactive form of protease domain modeled using template 3STI. B. Substrate binding site of active form of protease domain modeled using template 3STJ. C. The orientation and Cα distance between the catalytic triad molecules in the inactive form. D. The orientation and Cα distance between the catalytic triad molecules in the active form.

The Ser53 present in Loop L1 of VCO395_1035 was found to be conserved with the Loop L1 of the DegQ protease domain template (PDB ID: 3STI). This conserved Ser53 was retained in active site of the protease domain of VCO395_1035. The residues His50 and Leu52 of active site loop L1 were lined up in a one side of the active-site cleft, forming the catalytic triad with Ser12 of loop LD (Figure 5). To examine the role of catalytic triad, the 3D-model of protease domain was generated by utilizing inactive form of DegQ protease domain template (PDB ID: 3STI). On comparing Cα distance between the catalytic triad molecules (Table 1) and active site arrangement of active and inactive form of the protease domain (Figure 6A–D), it was clear that the predicted Ser12-His50-Leu52 catalytic triad had an important role in the Oxyanion hole formation, and Ser53 rearrangement in protease active site directly exposed it to substrate molecule.

**Table 1:** Comparison of the catalytic triad residues and active site arrangement of active and inactive form of the protease domain.

Model	Template	Catalytic Triad residue	Conformation	Cα distance of triad (Å)		
				S-H	H-L	L-S
VCO395_1035 (protease)	3STI	Ser12, His50 and Leu52	Inactive	5.6	5.52	7.4
VCO395_1035 (protease+PDZ1)	3STJ	Ser12, His50 and Leu52	Active	4.61	5.12	5.18

### Basic Trimeric Unit and Activation Mechanism

It is recognized that DegP of *E. coli* undergo substrate induced oligomer formation and the activation is of vital importance for HtrA protease regulation [45], [46]. Recently the same mechanism was observed in the DegQ [17]. It is known that Protease and PDZ domain has the important role in the oligomerization. The all HtrA protease exhibit a similar domain architecture and share a common trimeric building block, which are controlled by the conserved activation mechanism [47]. It had been observed that in presence of substrate formation of higher order 12-meric particles takes place while in absence of substrate, trimer formation occurred. Moreover, absence of the PDZ1 domain resulted in protease domain capable of forming basic timer unit, but was unable to perform the proteolytic activity and underwent higher order oligomerization. It was also observed that only PDZ1 was essential to couple substrate binding with the formation of proteolytically active higher order DegQ oligomers.

In the present study the predicted 3D-model of VCO395_1035, contained the Protease+PDZ1 domain, the essential mechanistically important activation loop and structural motif important in the oligomerization of HtrA family protein. These were well retained in predicted 3D model of VCO395_1035. Hence it could be hypothesized that VCO395_1035 may undergo higher order oligomerization and similar activation mechanism, as found in highly conserved DegP/DegQ HtrA protease.

**Figure 7:**
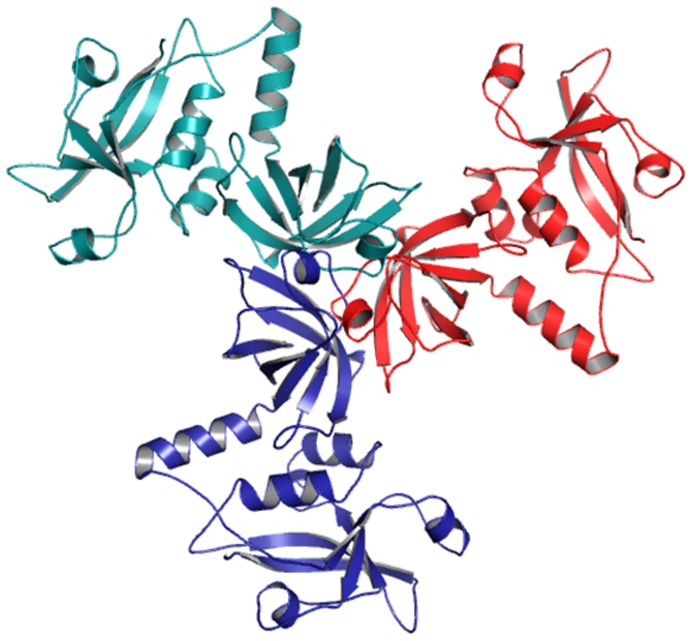
Basic Trimer Unit. Basic trimeric unit of the hypothetical protein (VCO395_1035) was formed by superimposing the monomer into the trimeric unit of template (PDB ID: 3STJ chain A, B & C).

To study the activation mechanism, the basic trimeric unit of VCO395_1035 was built. Basic trimeric unit (Figure 7) was formed by the docking the monomer into the trimeric unit of template (PDB ID: 3STJ chain A, B & C). The spatial arrangement of trimer of VCO395_1035 resembled a planar triangle with centered protease and PDZ1 domains at the vertices. The peripheral PDZ1 domains contacted with each other through HtrA signature motif IC which was essential for higher order oligomer formation by mediating contact between juxtaposed trimers [46], [48]. The interaction clamp comprised hydrophobic region residue 127–147 among which Ser129, Phe136, Leu140, Val142, Ala146 and Phe147 were conserved.

**Figure 8:**
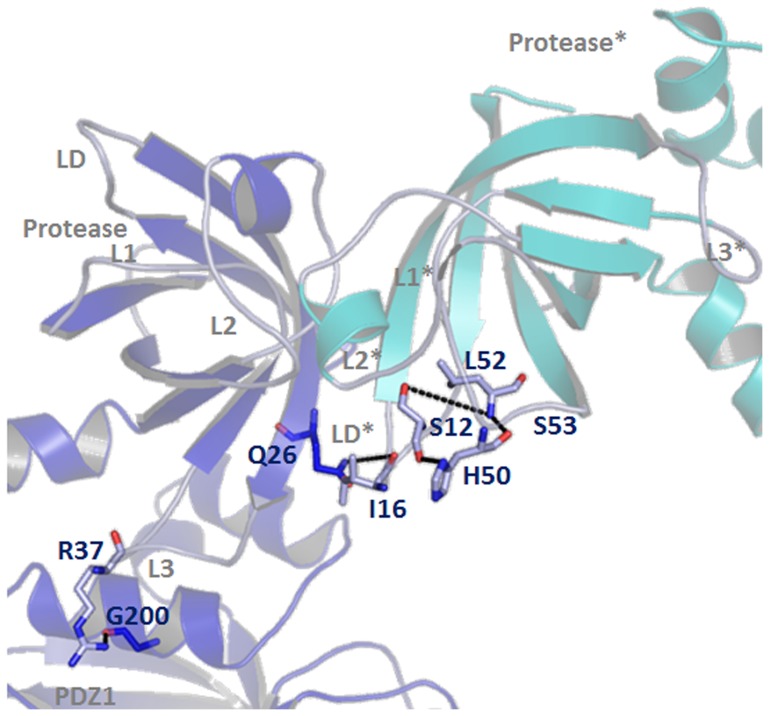
Mechanism of Protease activity. Illustration of the activation mechanism: PDZ1→L3→LD/L1/L3. The R37 of loop L3 interact with G200 of PDZ1 domain which allows Q26 of the loop L3 to interact with the residue I16 of loop LD in the adjacent protease shown in * mark. This may induce remodeling of the proteolytic sites and functional catalytic triad set up between S12 of loop LD and H50 & L52 of loop L1.

The activation of HtrA protease is known to be reversible process that could be triggered by distinct molecular signals. In DegS the substrate protein RseA signals the folding stress which are recognized and bound by the PDZ domain which are capable of inducing the rearrangement of sensor loop L3 which in turn re-modulate the activation domain into its functional state to cleave the substrate protein [49]–[51]. In DegP, substrate binding to the first PDZ1 domain induces the oligomer conversation from DegP6 to DegP12 and DegP24. This led to a repositioning and immobilization of the PDZ1 in such a way so as to induce rearrangement of loop L3 and perform protease activity [47], [48]. Similar mode of activation mechanism, as presumed for DegQ upon peptide binding to PDZ1, induces rearrangement of the protease loop L3 and stimulate the protease activity by activating the formation of catalytically active higher order oligomers [17]. The DegP and DegQ indicate the preserved intramolecular PDZ1→L3→LD/L1/L3 signaling constituent in regulating HtrA protease activity in both L2- and 24-meric HtrA oligomers [17]. To explore whether a similar PDZ1→L3→LD/L1/L3 protease activation cascade and molecular interplay between loop L3 and PDZ1 domain occured in predicted 3D-model of VCO395_1035, the monomer and basic trimeric unit was scrutinized. Interestingly, it was observed that there was the flip in the position of Arg and Gly residue (In the DegQ Arg302 of PDZ1 form a hydrogen bond with carbonyl oxygen of Gly171 in loop L3). In the 3D-model of VCO395_1035, Arg37 in the loop L3 formed the hydrogen bond with carbonyl oxygen of Gly200 in α7-helix of PDZ1 domain (Figure 8). The R37 of loop L3 interact with G200 of PDZ1 domain allowing Q26 of the loop L3 to interact with the residue I16 of loop LD in the adjacent protease. This may induce remodeling of the proteolytic sites and functional catalytic triad set up between S12 of loop LD and H50 & L52 of loop L1 (Figure 8). Hence the predicated model of VCO395_1035 indicated the preservation of intermolecular PDZ1→L3→LD/L1/L3 signaling event along with set up of catalytic triad. It was further hypothesized that like HtrA protease (DegP, DegQ), the loop L3 served as a molecular switch in regulating higher order oligomerization.

### Subcellular Localization

The subcellular localization of VCO395_1035 was predicted using CELLO, an approach based on a two-level support vector machine (SVM) system [32], [33]. The CELLO output gave significant reliability for outer membrane (1.493), periplasmic (1.477) and extracellular (1.426). SignalP [52] predicted it as a non-secretory protein. Localization study using the HSLpred [31] and the SubLoc v1.0 servers [34], both predicted it to be a periplasmic protein. This may be because of the fact that the PDZ domains of DegP proteins have been observed to be crucial for membrane localization [53]–[56]. Further, the lysine residues on the surface of PDZ domains in DegP has been reported to be essential for the lipid membrane attachment [48]. The presence of the lysine and arginine residues on the PDZ domain of the modeled 3D structure of the protein VCO395_1035 indicated that it may interact with the lipid membrane.

**Figure 9:**
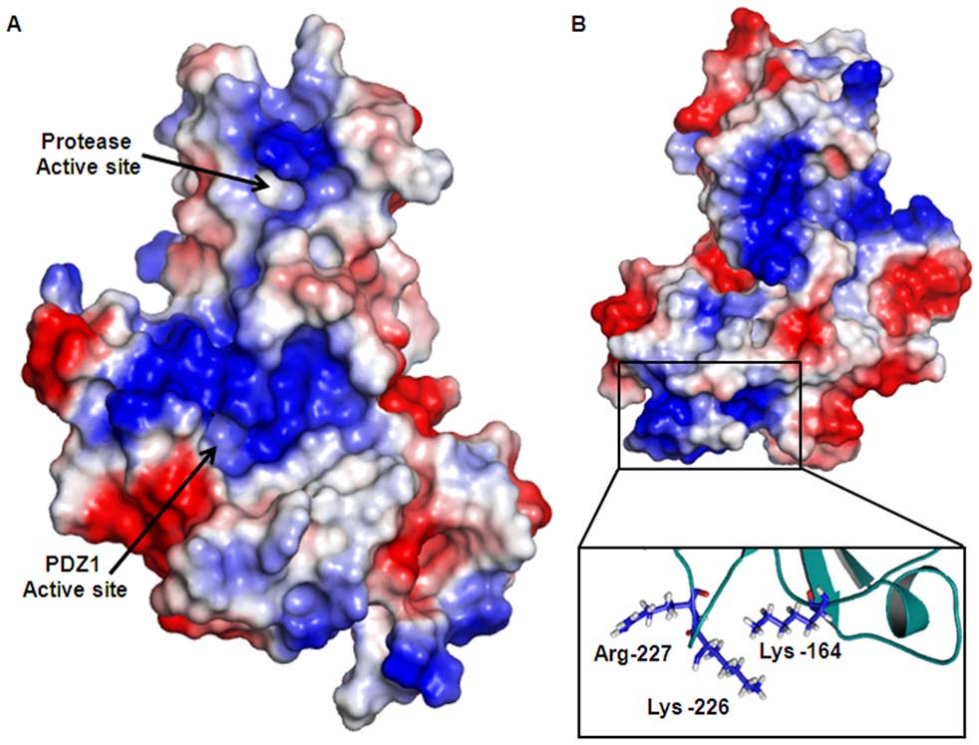
Surface electrostatic potential calculated by PyMOL. The positive charge shown in Blue and negative charge shown in Red. A. The active site of protease and PDZ1 domain showing more positively charge and less neutral environment. B. The outer surface of VCO395_1035 showing the blue patches spreads all over the molecule. The positive charge shown in rectangular frame is aggregated from Argine and Lysine residue. The inset shows orientation of Arg-164 & 227 and Lys-226 residue in cartoon representation, which are predicted to interact with outer membrane.

It has been well studied in *Escherichia coli* that the functionality of the three HtrA proteases (DegP, DegQ, and DegS) is regulated in the Cytoplasmic membrane via one transmembrane segment. To test this hypothesis and explore if the modeled 3D structure of VCO395_1035 might interact with the lipid membrane, the electrostatic potential of VCO395_1035 was generated by using in PyMOL [57]. The active site had greater positive charge than neutral charge. This mixed electrostatic potential around the active site of Protease domain and PDZ1 domain were assumed to be essential for attraction of C-terminal of substrate which is negatively charged (COO^−^) and to perform proper binding of substrate into the active site (Figure 9A). The outer surface of the PDZ1 domain showed strong positive charge (Figure 9B) originating from the cluster of lysine and arginine residues, which might be the candidate site for membrane attachment [48]. The residue Lys-164, Lys-226 and Arg-227 were forming positive electrostatic potential as shown in the inset of Figure 9B.

## Discussion

In the present study a hypothetical protein VCO395_1035 was identified by *Design-Island* as a part of horizontally acquired region in the large chromosome of *V. cholerae* O395. This gene showed a strong homology with conserved phage protein. To determine the possible function this protein, comparative protein structure modeling was done.

The study showed that the protein VCO395_1035 had >30% sequence similarity to protease+PDZ1 domain of HtrA DegQ, however there was lack of the initial residues containing the LA loop in VCO395_1035 when aligned with DegQ.

In the DegQ protein of *E. coli* the function of the LA loop is still elusive. The LA loop and the subsequent loops contain two of the catalytic triad residues His82 and Asp112 (3STJ). However, proteins with mutations to the catalytic triad have been reported to be present in many enzyme families. It has been estimated that up to 15% of the members of all encoded enzyme families may have lost their catalytic activity [58]. In many cases the inactive homologues are believed to have acquired alternative functions, such as competing with and antagonizing the active proteases, or otherwise regulating their function. Wrase et.al [59], recently showed in their study of *Legionella* HtrA DegQ homologue, deletion of LA loop did not affect formation of 12-mers in solution or proteolytic activity. There are several proteolytically active unconventional serine protease which having “serine only” configuration in the active site such as *Ochrobactrumanthropi* L-aminopeptidase D-Ala-esterase/amidase [60], *E. coli* Penicillin G acylase precursor [61], [62], Glutaryl 7-aminocephalosporanic acid acylase precursor (GCA precursor) [63].

In the predicted 3D-model of VC1035_protPDZ1, simplest catalytic centre serine was discovered which is conserved and is utilized for proteolysis. Unlike a conventional catalytic triad which is usually composed of a Ser, His and an Asp residue the presence of another functionally active catalytic triad gives insight to the understanding of proteolytic mechanism and how serine protease preserved their mode of action.

The HtrA homologue from *E. coli* are under control of substrate-induced oligomer conversion and protease activation, irrespective of the presence of one or two PDZ domains [17], [45], [48]. Recently revealed crystallographic structure of DegQ and DegP with higher order oligomers suggested that signaling cascade leading to protease activation of 12- and 24-mer HtrA complex was highly conserved and depended on precise positioning of PDZ1 domain upon substrate engagement [17]. The present study revealed one type of serine protease homologue whose active site arrangements allowed these proteases to work in different environments of the host. Our homology modeling study and result analysis indicated that VCO395_1035, which has been annotated as a hypothetical protein, is predicted to be an unconventional serine protease of atypical HtrA homologue performing similar function.

## Materials and Methods

### Acquisition of Sequences

The complete genome sequences of *V. cholerae* O395, the O1 classical strain of Ogawa serotype isolated in 1964 from India was considered for the present study. The chromosomal sequences of the organism were downloaded from the ftp server of NCBI database (http://www.ncbi.nlm.nih.gov/genomes/lproks.cgi).

### Detection of putative GI using *Design-Island*

The program *Design-Island* developed in-house [27] was used for the identification of the putative GIs in the chromosomes of *V. cholerae* O395. *Design-Island* searches for islands in a prokaryotic chromosome using a probing window of varying size that slides over the entire chromosome. It uses an algorithm which is an unsupervised one and applies Monte Carlo's statistical test on randomly selected segments on a genome. Precise statistical distribution theory then determines the reliable P-values for making the decision.

The program *Design-Island* runs in two phases, namely *first phase* and *refinement phase*. In the *first phase*, it identifies islands at different locations of the chromosome and to determine the stretches of those islands, and carries out statistical analysis using a probing window. This leads to the identification of some ‘putative GIs’ having varying sizes and locations in the chromosome that are identifiable with P-values generated using Monte-Carlo tests carried out at variable locations of the probing window with a fixed size. In the first phase, *Design-Island* was run using P_0_ = 0.05, word size of 4 and initial window size of 5000 with consequent window increment of 500. 200 randomly selected fragments were tested for each window with a sliding window 500.

Following the *first phase*, *refinement phase* commences which takes random samples of genomic segments excluding the regions detected in the *first phase*. Some of the putative GIs identified in the *first phase*, are further refined into smaller segments containing horizontally acquired genes in the *refinement phase*. In this phase *Design-Island* was run with the same parameter values as used in the *first phase*, except for the initial window size, which was reduced to 2000 and the sliding window increased to 1000. The statistical analysis in the *refinement phase* is similar to that used in the *first phase* except the P_0_ was set to 0.001. The results thus obtained were tabulated using customized Perl scripts where the cut-off E-value was set to 0.001.

### Localization Study

The subcellular localization of the target protein was predicted using HSLpred [31], CELLO [32], [33] (http://cello.life.nctu.edu.tw/), SubLoc v1.0 [34] and SignalP [52].

### Template Selection for homology

The template selection for the homology modeling of the target protein was performed by submitting the amino acid sequence of the target protein in BLAST [64], [65], PBD-BLAST [66], SWISS-Model [67], CPH models [68], 3D-JIGSAW [69], ESyPre3D [70], Geno3D [71], HHpred [72] and ModWeb servers [35].

### Alignment Study

The alignment study was performed by using CLSTALW [73], FUGE [74], T-Coffee [75] and MUSCLE [76], [77] servers. During the alignment, the insertion of gaps were allowed in the region of final alignment in such a way that the secondary structure was not disturbed and first 241 amino acid residues of target were threaded into the Protease+PDZ1 domain (residue 136–334) template structure.

### Model Construction and Validation

The three-dimensional structure of the target protein was performed using a restrained-based approach in MODELLER9v6 [36], [78]. FALC-Loop: Protein Loop Modeling Server was used for predicting the local structure of loops [40]. The final Deviations in the protein structure geometry was regularized by energy minimization with the GROMOS96 force field [38] using Deep View [39]. The final model was validated by using PROCHECK [42] and TM-align [43].

### Docking Study

The docking was performed using the Hex 5.0 software [44], with the reference of template complex with the substrate molecule. The electrostatic potential calculation, model visualization and image generation was performed using the PyMOL software [57] (www.pymol.org).

## Supporting Information

Figure S1 **Algorithmic flow-chart for generation of the circular map indicating GIs on the chromosome.**
(TIF)

Figure S2 **Circular map representing an individual chromosome of** ***V. cholerae*** **O395 representing the region covered by the predicted GI.** The map shows two circles representing the putative regions of the same chromosome in separate phases. The inner circle with regions marked in blue represents the predicted regions obtained in the first phase of the run by *Design-Island*. The outer circle with red regions represents the putative regions as predicted by *Design-Island* in the refinement phase or the second phase. *V. cholerae* O395 large chromosome. *V. cholerae* O395 small chromosome.
(TIF)

Figure S3 **Ramachandran plot for predicted 3D model of VCO395_1035 generated by PROCHECK.** Most favored regions indicated in red, additional allowed in yellow, generously allowed in light yellow and disallowed regions indicated in white fields.
(TIF)

Figure S4 **Superposition of 3D-model of VCO395_1035.** The superimposition model generated by PyMOL, where VCO395_1035 is shown in pink and the template 3STJ in blue.
(TIF)

Table S1 **PROCHECK report for the final model of VCO395_1035.**
(DOC)

Table S2 **Characterization of 3D-model of VC0395_1035.**
(DOC)

Table S3 **Residues involve in the active site formation.**
(DOC)

Table S4 **Residues involve in the substrate binding.**
(DOC)
