# Supplementary figures and images for: In-Silico Structural and Functional Characterization of a V. cholerae O395 Hypothetical Protein Containing a PDZ1 and an Uncommon Protease Domain
Source: PLoS One. 2013 Feb 18;8(2):e56725. doi: 10.1371/journal.pone.0056725 (PMC3575494; doi:10.1371/journal.pone.0056725)

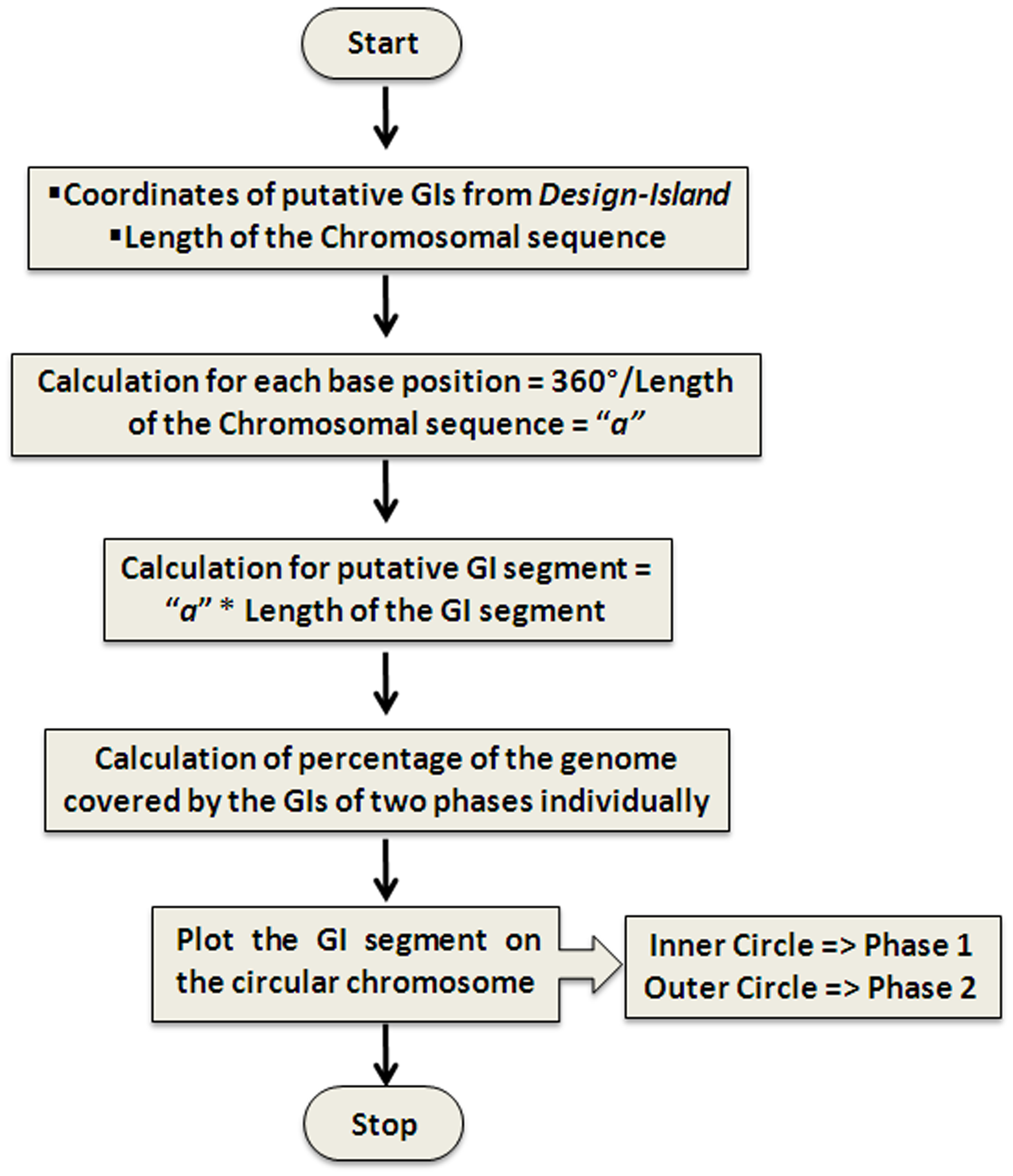

Supplement: Figure S1 — Algorithmic flow-chart for generation of the circular map indicating GIs on the chromosome. (TIF) [file pone.0056725.s001.tif]

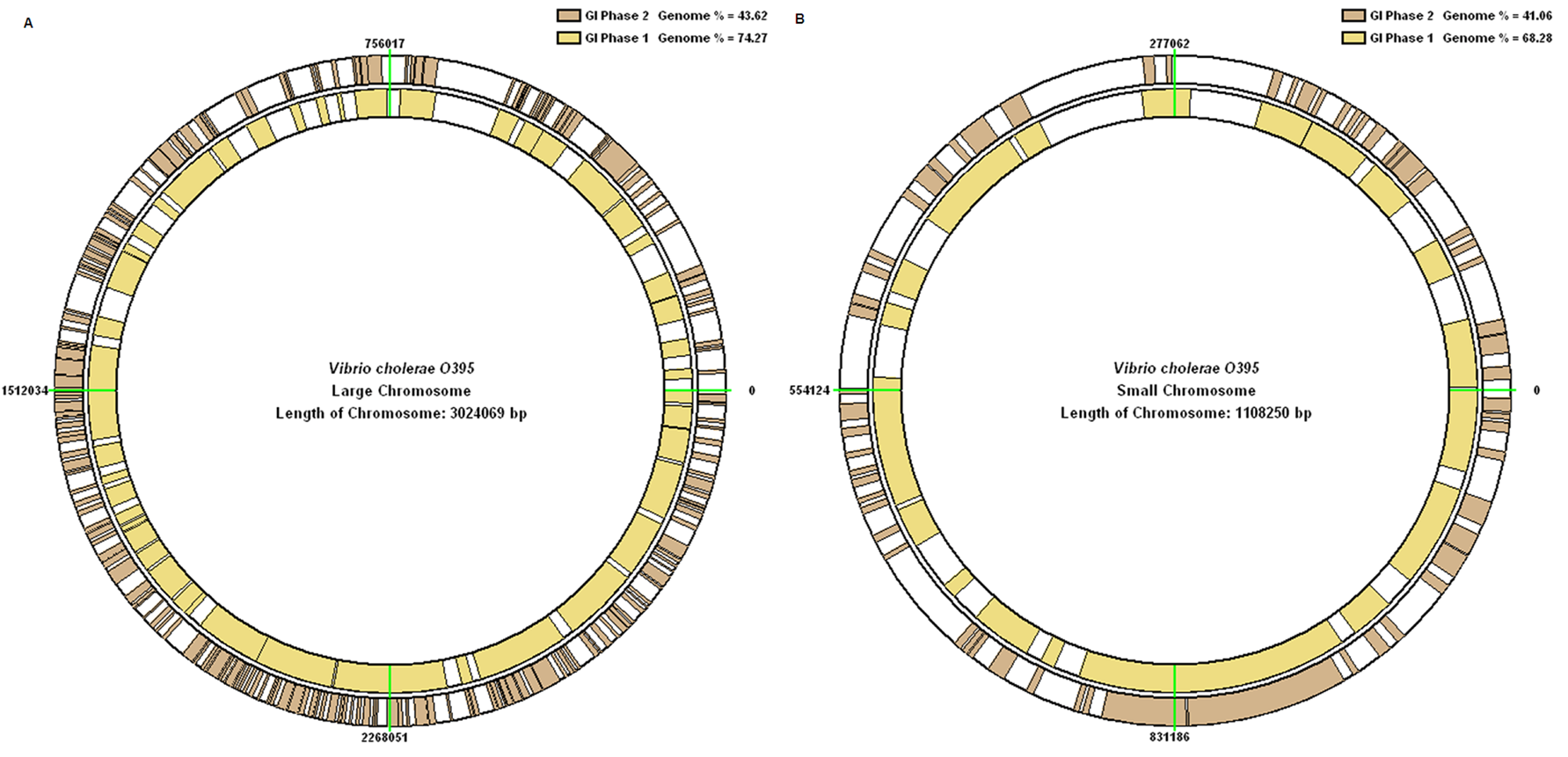

Supplement: Figure S2 — Circular map representing an individual chromosome of V. cholerae O395 representing the region covered by the predicted GI. The map shows two circles representing the putative regions of the same chromosome in separate phases. The inner circle with regions marked in blue represents the predicted regions obtained in the first phase of the run by Design-Island. The outer circle with red regions represents the putative regions as predicted by Design-Island in the refinement phase or the second phase. V. cholerae O395 large chromosome. V. cholerae O395 small chromosome. (TIF) [file pone.0056725.s002.tif]

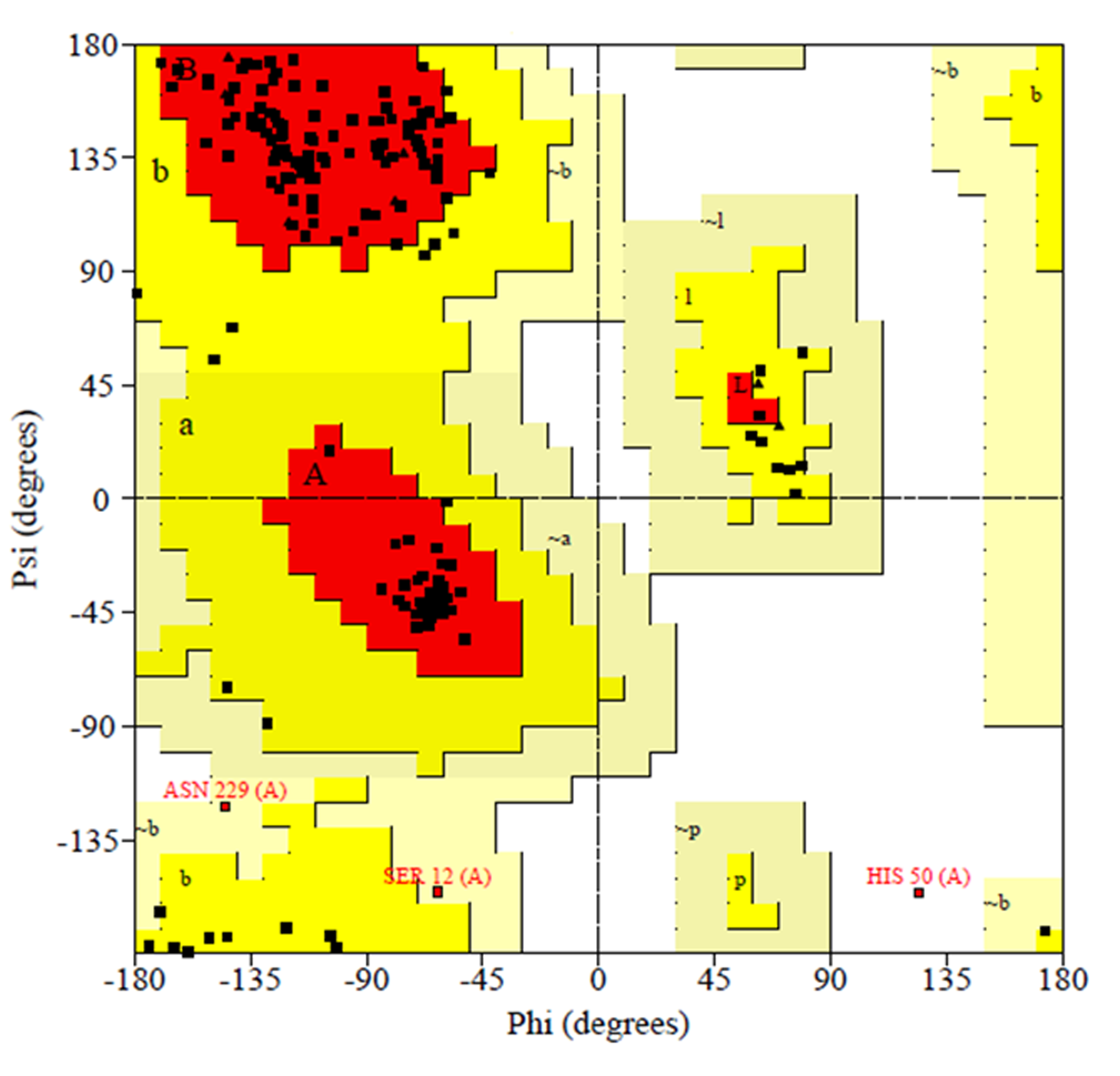

Supplement: Figure S3 — Ramachandran plot for predicted 3D model of VCO395_1035 generated by PROCHECK. Most favored regions indicated in red, additional allowed in yellow, generously allowed in light yellow and disallowed regions indicated in white fields. (TIF) [file pone.0056725.s003.tif]

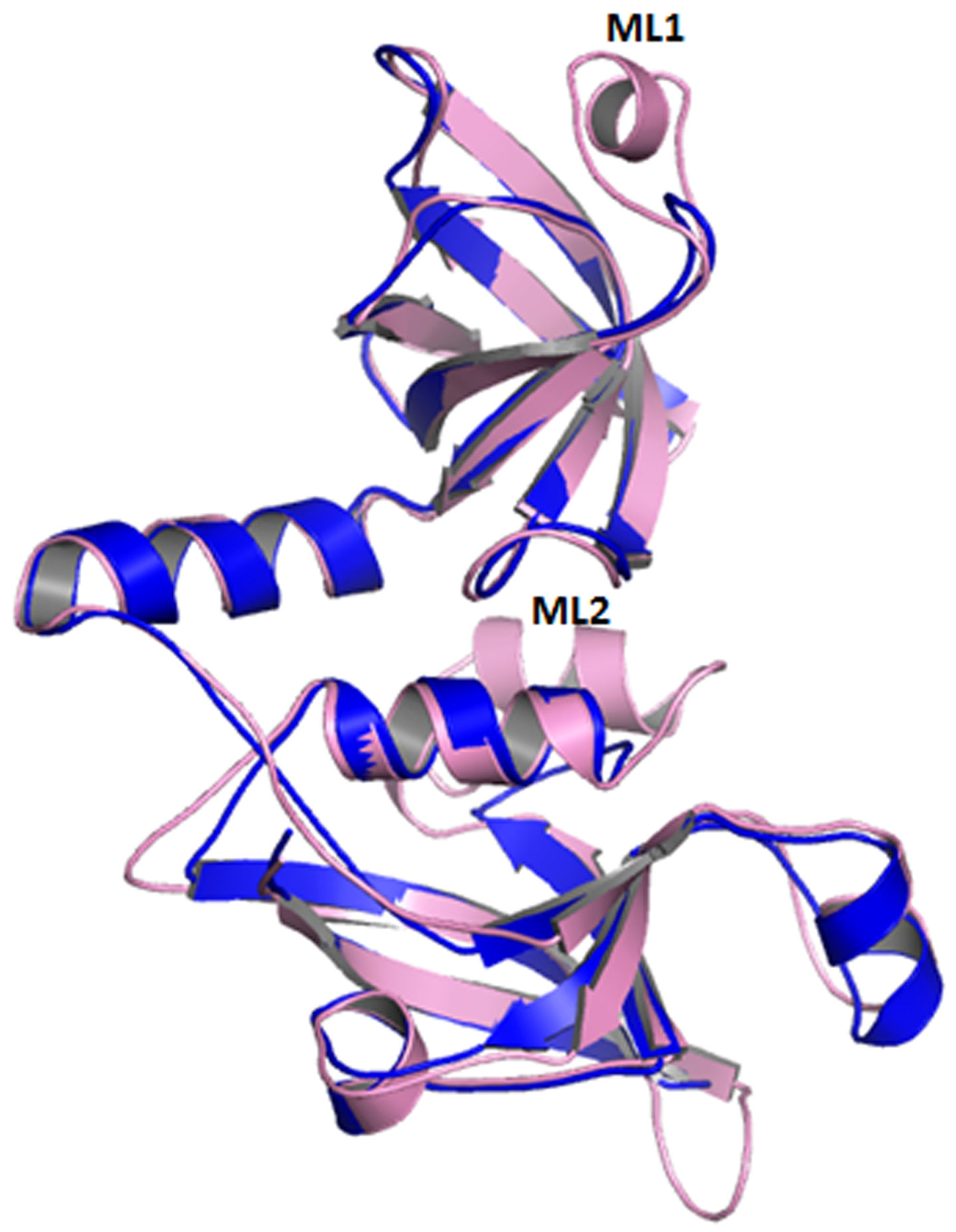

Supplement: Figure S4 — Superposition of 3D-model of VCO395_1035. The superimposition model generated by PyMOL, where VCO395_1035 is shown in pink and the template 3STJ in blue. (TIF) [file pone.0056725.s004.tif]
