# Supplementary material for: In-Silico Structural and Functional Characterization of a V. cholerae O395 Hypothetical Protein Containing a PDZ1 and an Uncommon Protease Domain
Source: PLoS One. 2013 Feb 18;8(2):e56725. doi: 10.1371/journal.pone.0056725 (PMC3575494; doi:10.1371/journal.pone.0056725)
Supplement: Table S1 — PROCHECK report for the final model of VCO395_1035. (DOC) [file pone.0056725.s005.doc]

Table S1 PROCHECK report for the final model of VCO395_1035

| **Ramachandran plot** | 82.7 % core | 15.7 % allowed | 1.1 % generous | 0.5 % disallowed |
| --- | --- | --- | --- | --- |
| **Main-chain parameters** | 6 better | 0 inside | 0 worse |  |
| **Side-chain parameters** | 5 better | 0 inside | 0 worse |  |
| **G-factors** | Dihedrals: -0.17 | covalent: -0.41 | overall: -0.24 |  |
| **M/c bond lengths** | 98.8 % within limits | 2 % highlighted |  |  |
| **M/c bond angles** | 88.1 % within limit | 11.9 % highlighted |  |  |
| **Planar groups** | 100.0% within limits | 0.0% highlighted |  |  |
