# Supplementary material for: In-Silico Structural and Functional Characterization of a V. cholerae O395 Hypothetical Protein Containing a PDZ1 and an Uncommon Protease Domain
Source: PLoS One. 2013 Feb 18;8(2):e56725. doi: 10.1371/journal.pone.0056725 (PMC3575494; doi:10.1371/journal.pone.0056725)
Supplement: Table S2 — Characterization of 3D-model of VC0395_1035. (DOC) [file pone.0056725.s006.doc]

Table S2 Characterization of 3D-model of VC0395_1035

| **Domains** | **α-helix (AA)** | **β-sheet (AA)** | **Important Loops (AA)** |
| --- | --- | --- | --- |
| **Protease Domain** | α1 (82-86)^ML1^ | β1 (3-9) | LD (10-19) |
|  | α2-IDL- (98-111) | β2 (21-29) | L3 (30-39) |
|  |  | β3 (40-43) | L1 (44-53) |
|  |  | β4 (54-58) | L2 (74-81) |
|  |  | β5 (67-73) |  |
|  |  | β6 (93-97) |  |
| **PDZ1 Domain** | α3-IC- (131-136) | β7 (125-128) | CBL (119-122) |
|  | α4 (157-161) | β8 (146-150) |  |
|  | α5 (173-177) | β9 (168-171) |  |
|  | α6 (181-188)^ML2^ | β10 (214-221) |  |
|  | α7 (196-205) | β11 (229-238) |  |
