# Supplementary material for: In-Silico Structural and Functional Characterization of a V. cholerae O395 Hypothetical Protein Containing a PDZ1 and an Uncommon Protease Domain
Source: PLoS One. 2013 Feb 18;8(2):e56725. doi: 10.1371/journal.pone.0056725 (PMC3575494; doi:10.1371/journal.pone.0056725)
Supplement: Table S3 — Residues involve in the active site formation. (DOC) [file pone.0056725.s007.doc]

Table S3 Residues involve in the active site formation.

| **Active Site** | **Residue** |
| --- | --- |
| **Protease Domain** | Leu47, Gly48, Arg49, His50, Pro51, Leu52,Ser53, Leu70, Asn71, Gln72, Gly73 and Thr79. |
| **PDZ1 Domain** | Gly119, Gln120, Leu121, Tyr122, Ala123,Val124, Ala125, Ile126, Met127, Val128, Phe196, Ala197, Ala198, Gln199, Gly200, Ala201, Ile203, Ala204 |
