# Supplementary material for: In-Silico Structural and Functional Characterization of a V. cholerae O395 Hypothetical Protein Containing a PDZ1 and an Uncommon Protease Domain
Source: PLoS One. 2013 Feb 18;8(2):e56725. doi: 10.1371/journal.pone.0056725 (PMC3575494; doi:10.1371/journal.pone.0056725)
Supplement: Table S4 — Residues involve in the substrate binding. (DOC) [file pone.0056725.s008.doc]

Table S4 Residues involve in the substrate binding.

| **Active Site** | **Residue** |  |
| --- | --- | --- |
| **Protease Domain** | Ser53, Asn71, Gln72 ,Gly73,Thr74, Val75,Leu76 | |
| **PDZ1 Domain** | **P_0_-pocket:** Gly119, Gln120, Leu121, Tyr122, Ala123, Gly200, Ile203 and Ala204  **P_-2_-pocket:** Ala123, Val124, Ala125, Ile126 and Phe196 | |
